# Echoes of the Month

**Published:** 1924-10

**Authors:** 


### ECHOES OF THE MONTH.

### INQUEST FEES.

Dr. Edward Smith, coroner for North-East London, referred at a Hackney inquest to a recent decision of the London County Council that in future the fees paid to infirmary medical officers should be disallowed, on the ground that such fees were unauthorised under the Coroners' Act of 1887. In the event of the legal position being tested and upheld, he hoped there would be fresh legislation which would restore the fees. Not only that, but he hoped that at the same time the doctors of the voluntary hospitals would become entitled to the fees of which they had all along been deprived. If preference were to be given to any one class of doctor, surely it should be the medical officers of the voluntary hospitals, for their posts carried no salary, whereas the salaries of infirmary doctors could be assessed, and probably were assessed, with some regard to the number of inquests with which they were likely to be concerned. It was a mistaken policy to demand gratuitous work from skilled professional witnesses.

### SHOULD WE EAT FRUIT SKINS?

A West End physician told the *Westminster Gazette* recently that there are two schools of dietitians, who might be termed skin-eaters and non-skin-eaters. "The object of the latter," said the doctor, "is to insure that children's food should be as digestible as possible. They would not allow any indigestible things such as fruit skins. The other school emphasises the fact that the ordinary healthy person requires a certain amount of indigestible matter, otherwise the bodily functions are impaired." In his own opinion, the doctor said that, in the case of a child just recovering from an illness, with its stomach in a delicate condition, it was necessary to be very careful, and give only digestible food. Adults and bigger children in good health, however, should eat the skins of ripe fruit. The fruits he referred to were such as apples, pears, peaches, apricots and plums.

### PUBLIC HEALTH RHYMES.

Verses and pictures flashed by electric signs in the main streets form the latest development in the public health campaign. Bermondsey is responsible for this novel idea, and its deputy medical officer, Dr. Connan, has added to his duties the task of rhymester. First a picture of a decrepit old man walking with the aid of a stick is flashed on the screen, and then follows the explanatory verse :—

"Gums full of poison, teeth few and loose,
At forty lumbago, at fifty the screws."

Then, as a contrast and an example to be followed, appears a picture of a happy, healthy old man using a toothbrush. It is followed by the verse :—

"I am eighty to-day, yet from aches I am free ;
The toothbrush way is the way for me."

### WHERE BREAD IS REALLY THE STAFF OF LIFE.

Lieut.-Colonel H. Halliday, who practised for twenty years as a surgeon in Simla, writes in the "Lancet" that the natives of the Punjab, the north-west province of India, whose diet is mainly coarse wholemeal bread, seldom suffer from cancer. The diet of these people consists of lightly scorched unleavened bread, made from a dough of wholemeal stone-milled unsifted flour into cakes about the size and shape of our pancakes. This bread is eaten with däl, a sort of pulse, or with curried vegetables. A large quantity of cellulose is taken in the form of raw radishes, sugar cane and raw fruit. The peasants also drink quantities of milk. With them bread is the staff of life. They have magnificent teeth and are usually of fine physique.

### WEMBLEY SWITCHBACK AS A TONIC.

"In civilised countries," writes Dr. Ronald Macfie, "the stimulation of fear is too seldom provided. No normal man can take the great plunges on the switchbacks at Wembley without experiencing a certain amount of trepidation—I personally have taken the plunges, and I am not afraid to admit I was afraid—and the fear undoubtedly must stimulate the secretions of the thyroid, supra-renal, and pituitary glands, while the rush through the air must be as a draught to the vital furnaces. The courageous plunger steps out of the switchback car with wonderful subtle substances in his blood fitting him for battle or for flight. His blood pressure is higher ; his fuel supply is augmented ; his vital fires burn more brightly; and, even if he do not fight or take to flight, he is thoroughly 'bucked-up.' Probably that is why the switchback is so popular, and why men, women, and children enjoy being afraid even to the screaming point."

### SUCCESSFUL TREATMENT OF LEPROSY.

Successful treatment of a case of leprosy at the Royal Infirmary, Edinburgh, is described in the *Lancet* by Sir Norman Walker. The patient was a Russian Jewess, aged 70, who went to Scotland over 40 years ago. She had been married for 52 years, and has had two children, both of whom are alive and well. She was admitted to the hospital in August last year. After trying a treatment which caused great pain and could not be continued, Sir Norman tried injections with a vaccine prepared direct from tuberculous glands, which he had found effective in cases of lupus. Within three weeks improvement was noticeable, and it proceeded with accelerated strides until the whole eruption had practically disappeared.

### THE ISOLATION OF VITAMIN A.

The Chemical Society of Japan states that two Japanese chemists have successfully isolated what they believe to be Vitamin A. This is perhaps the most important of these mysterious substances now definitely known to exist. Its absence in food is associated with rickets in children and other diseases of nutrition. Hitherto nothing has been known concerning its chemical structure and very little about its properties. The Japanese chemists, however, have analysed the substance they obtained. Whether or not it is Vitamin A remains a question for further verification. A mouse at the point of death because of lack of Vitamin A was given minute quantities of the substance daily, and in ten days was restored to complete health.

### IS THE CLERK HEALTHY?

"A comparative estimate of the health of the clerk as a whole," writes Dr. Thomson, M.O.H. for Deptford, in "The World's Health," "can be made from a study of vital statistics. In England and Wales there are 180 occupational groups, and a comparative mortality figure for each group has been calculated for the ages between twenty-five and sixty-five years. At the healthy end of the table we find motor-car and motor-van drivers, gardeners, nurserymen, gamekeepers, agricultural labourers and clergymen, most of whom live more or less an open-air life. At the sick end of the table we find the general labourer, barmen, tin miners and file makers. The clerk stands midway in the list. He is pulled towards the healthy end by the insurance, bank, civil service and railway clerk, but the law clerk and the commercial clerk draw him back to halfway. The average layman would probably have placed the clerk higher, say fortieth or fiftieth, instead of eighty-seventh, as he actually is. This is because people regard the clerk as a black-coated gentleman whose vocation places him above the classes engaged in the turmoil and stresses of life. I am convinced that the clerk should, for his health's sake, receive a three weeks' minimum summer holiday in addition to the usual statutory holidays. He should not be haunted all his days by the fear of penurious old age. A compulsory pension scheme is essential."

### ETHER COCKTAILS.

The ether peril is declared by French physicians to be increasing at an alarming rate. As the restrictions upon the use of such drugs as cocaine and morphia have become more difficult to evade, and supplies of these poisons consequently harder to obtain, large numbers of drug slaves have sought a substitute in the use of ether, which is cheap and as easy to obtain as methylated spirits. They have found that, when inhaled in small doses, ether gives a pleasant sensation of exultation and well-being, while larger doses have a narcotic effect. Many people have contracted the habit of sipping it slowly, when it has an effect very similar to that of cocaine, and there has sprung up in Montmartre quite a lucrative traffic in pocket-flasks of ether, which are freely sold in night resorts at 20 francs each—a price which yields 15 francs profit to the vendor. Ether cocktails, which consist of a teaspoonful of ether in a glass of champagne, are also being dispensed.

### LITERATURE FOR THE BLIND.

The publication of books and periodicals in embossed types has always been one of the most important branches of the work of the National Institute for the Blind. Since 1916 over 1,700,000 publications have been issued, including books of all kinds and many periodicals. Two newspapers are issued, the *Braille Mail* and the *Moon*—the latter being the smallest newspaper in the world, consisting of only 800 to 850 words per issue. There is also a monthly magazine, *Progress*, of general interest; the *School Magazine*, with original competitions; a literary review, a musical monthly, and a devotional magazine.

### A HEALTH GAME.

Forty thousand British school-children, members of junior Red Cross societies throughout the country, are being taught the principles of hygiene by means of a new Red Cross "Health Game." Each child has to observe six rules of health printed on a chart and signed and countersigned daily by children and parents. These rules are: Wash your hands before meals; sleep with the window open; brush your teeth; bath at least once a week; drink only milk or water; play one game in the open air every day. The game is being organised all over the world, and in Albania recently it was the means of inducing a tribal chieftain, who washed seldom and combed never, to shave his head completely because his daughter, who had been to a health centre, refused to kiss him until he did. In Bulgaria the rule relating to the weekly bath had to be amended to "a bath every month," as the officials considered it advisable to bring it within the bounds of practicability.

### EARLY DAYS OF THE ROYAL HUMANE SOCIETY.

The 150th anniversary of the Royal Humane Society occurs this year. Originally the methods of the society were confined to the recovery of the drowned. Later on they were extended to every known species of apparent sudden death, and even suicide. In this connection it is, by the way, noteworthy that in 1794 Dr. Hawes, one of its founders, described suicide as "a most heinous crime, said by foreigners to be almost peculiar to this country." The success of the society rapidly grew, owing to the large number of cases successfully handled, though the early efforts of the promoters, while earnest and sympathetic, afford amusing reading to the scientific intellects of to-day.

### NURSING HOME FOR CIVIL SERVANTS.

It is proposed that a nursing home should be instituted to provide Civil Servants with the best possible surgical and medical treatment, at a reasonable cost, for themselves and their near relatives. It is thought that, in view of the numbers of Civil Servants whom such a home would serve, the maintenance expenditure could be met fairly easily by a uniform subscription, which should not be beyond the means of even the lower paid grades. As an alternative to the provision of a special nursing home, it has been suggested that, by the institution of a Civil Service Provident Hospital Association, arrangements might be made with existing voluntary hospitals to secure the provision of beds in private patients' wards.

### HEALTH VISITORS IN SCOTLAND.

Under the above heading the Scottish National Association of Health Visitors, Women Sanitary Inspectors, and School Nurses has recently issued a handbook giving particulars of the conditions for the certification and registration of Health Visitors, of a model course of training, of salaries of Health Visitors, and of Maternity Service and Child Welfare Schemes. It gives details of the welfare work carried on in certain districts by Women Sanitary Inspectors, Health Visitors, Tuberculosis Nurses, and Venereal Disease Nurses. The recent discussions in this country with regard to the Health Visitor's salary make it worthy of notice that in Scotland salaries vary from £100 to £300 without bonus and uniform.

